# Genome-wide identification and functional characterization of the *CP12* gene family in cotton reveals its critical role in heat stress response

**DOI:** 10.3389/fpls.2025.1707567

**Published:** 2025-10-30

**Authors:** Chao Li, Shuguang Li, Juan Xu, Ziling Han, Wenlong Li, Yanhai Zhao, Yanqin Wang

**Affiliations:** Xinjiang Production & Construction Corps Key Laboratory of Protection and Utilization of Biological Resources in Tarim Basin, College of Life Science and Technology, Tarim University, Alar, Xinjiang, China

**Keywords:** cotton, CP12 gene family, genome-wide identification, heat stress, physiological response

## Abstract

**Introduction:**

Calvin Cycle Protein 12 (CP12) is a key regulator of the Calvin-Benson-Bassham (CBB) cycle that mediates CO₂ assimilation through dark/light modulation. Beyond its canonical role, emerging evidence indicates that CP12 may also function as a molecular chaperone and participate in plant stress responses. However, its gene family characteristics and roles under heat stress remain unclear in cotton.

**Methods:**

We performed a genome-wide identification and characterization of the *CP12* gene family in four cotton species (*Gossypium hirsutum, G. barbadense, G. arboreum, and G. raimondii*). Phylogenetic classification, conserved motif analysis, gene structure, synteny, and promoter cis-element analyses were conducted. Transcriptome datasets from flowers, leaves, and buds under heat stress were analyzed to determine expression patterns, and these were further correlated with physiological indicators.

**Results:**

A total of 11, 10, 5, and 4 CP12 genes were identified in *G. hirsutum, G. barbadense, G. arboreum*, and *G. raimondii*, respectively. Phylogenetic analysis grouped them into three clades (I–III), supported by conserved motif and structural features. Synteny analysis indicated that whole-genome and segmental duplications were the primary drivers of expansion. Promoter analysis revealed enrichment of stress-responsive elements. Expression profiling showed clade-specific divergence: Clade I genes were strongly induced by heat stress, with Ghir_CP12_10 displaying ~10-fold upregulation in flowers, while Clade II genes were generally downregulated. These expression trends were associated with physiological changes, including reduced net photosynthetic rate and elevated malondialdehyde, catalase, and peroxidase levels.

**Discussion:**

Our findings demonstrate that the cotton CP12 gene family has undergone functional divergence. Clade I members act as positive regulators of thermotolerance, potentially stabilizing photosynthetic complexes and protecting enzymes from oxidative damage under heat stress. This study provides new insights into the evolution and function of CP12 genes and establishes a foundation for future functional validation and breeding of heat-tolerant cotton varieties.

## 1 Introduction

Cotton (*Gossypium* spp.) is a globally important economic crop and a significant source of natural fiber. However, with changing climatic conditions, its yield is increasingly threatened by heat stress (Sheri et al., 2023). High temperatures during flowering and boll development can reduce pollen viability and increase boll abscission, ultimately leading to substantial yield loss (Li et al., 2024). Moreover, heat stress adversely affects multiple physiological processes, particularly photosynthesis. Elevated temperatures hinder the repair of Photosystem II (PSII), disrupt the CBB cycle, and promote the accumulation of reactive oxygen species (ROS), which cause oxidative damage to chloroplast membranes and photosynthetic enzymes (Marri et al., 2014; Gururani et al., 2015).

CP12 is an intrinsically disordered protein (IDP) (Gérard et al., 2022a). It is found widely across photosynthetic organisms and primarily resides within chloroplasts. It acts as a key regulator of photosynthetic carbon metabolism, particularly in modulating the activity of Calvin cycle enzymes (Gontero and Maberly, 2012). In its reduced state, CP12 maintains a flexible, unfolded conformation, whereas oxidation induces the formation of disulfide bonds between its N- and C-termini, resulting in a partially ordered structure (Del Giudice et al., 2023). The C-terminal domain mediates the assembly of a ternary complex of approximately 640 kDa, composed of glyceraldehyde-3-phosphate dehydrogenase (GAPDH), CP12, and phosphoribulokinase (PRK) (Marri et al., 2005). This reaction is exothermic and thermodynamically favorable, producing a compact “hollow rhombus” structure that prevents protein aggregation and protects catalytic sites from inactivation (Marri et al., 2008).

Beyond its regulatory role in the CBB cycle, CP12 also exhibits cytoprotective effects under abiotic stress conditions. In its oxidized state, CP12 can directly protect GAPDH from oxidative damage (Erales et al., 2009). In *Arabidopsis thaliana*, knocking out the *CP12* gene reduces PRK protein abundance without affecting transcript levels, indicating post-transcriptional regulation (Elena López-Calcagno et al., 2017). Under cold stress, low temperatures inhibit entirely the dissociation of the CP12/GAPDH/PRK complex, thereby suppressing the enzymatic activities of both PRK and GAPDH (Teh et al., 2023). Conversely, overexpression of CP12 in the tropical legume *Stylosanthes guianensis* increases biomass by approximately 30% under low temperatures (Gérard et al., 2022b). At the same time, the total loss of CP12 in *Chlamydomonas reinhardtii* leads to the up-regulation of 13 ROS-scavenging proteins and six molecular chaperones, underscoring its multifaceted role in stress protection (Gérard et al., 2022a).

Extensive research in model organisms has elucidated the canonical functions of CP12; however, its evolutionary adaptations are likely to vary across lineages. In diatoms, for example, CP12 lacks the conserved C-terminal cysteine pair (Groben et al., 2010). In cyanobacteria, it does not form the traditional ternary complex with GAPDH and PRK. Instead, it interacts with a cystathionine β-synthase (CBS) domain–containing protein, suggesting an alternative role in sulfur metabolism (Hackenberg et al., 2018). Compared to *Chlamydomonas reinhardtii*, *A. thaliana* CP12 exhibits greater structural disorder in its oxidized state, which may enhance its flexibility and redox responsiveness (Del Giudice et al., 2023). However, CP12 remains poorly studied in non-model plant species, particularly in allotetraploid cotton, where gene duplication and subgenome divergence may have promoted functional diversification.

In this study, we conducted a comprehensive genome-wide identification and characterization of the *CP12* gene family across four *Gossypium* species. We analyzed their phylogenetic relationships, structural conservation, cis-regulatory elements, and expression profiles under field-based heat stress. By integrating transcriptomic and physiological data, we aimed to elucidate the evolutionary divergence and potential functional specialization of *CP12* genes in cotton’s response to heat stress. Our findings provide novel insights into the molecular basis of thermotolerance, laying the groundwork for future functional validation and the development of heat-resilient cotton cultivars.

## 2 Materials and methods

### 2.1 Genome-wide identification of the *CP12* gene family

The reference genome sequences, along with their corresponding protein sequences and annotation files (GFF3 format), for four cotton species, the allotetraploids *G. hirsutum* (AD1, ‘ZM113’ T2T genome CRI_v1.0) (Hu et al., 2025) and *G. barbadense* (AD2, ‘3-79’ genome HAU_v2_a1) (Wang et al., 2019), and their diploid progenitors *G. arboreum* (A1, ‘SXY1’ genome HAU_v2) (Wang et al., 2022) and *G. raimondii* (D5, ‘Grai D502’ genome HAU_v1) (Wang et al., 2021), were obtained from the CottonGen database (https://www.cottongen.org/).

To comprehensively identify all members of the *CP12* gene family, an integrated strategy employing both BLAST and Hidden Markov Model (HMM) searches was implemented. For the BLASTP approach, protein sequences of three previously characterized *AtCP12* genes (AT1G76560.1, AT2G47400.1, AT3G62410.1) sourced from TAIR (https://www.arabidopsis.org/) were used as queries to search against the *Gossypium* proteomes with an E-value cutoff of 1e-10 (Chen et al., 2017). Concurrently, an HMMER search was conducted using the PF02672 (CP12 domain) profile obtained from InterPro (https://www.ebi.ac.uk/interpro/entry/pfam/PF02672/) to scan the same proteomes via the HMMER package embedded in TBtools software (v2.333) with an E-value cutoff of 1e-10 (Chen et al., 2023). Candidate sequences identified from both methods were merged, and the intersection was retained to enhance reliability. Finalized genes were systematically renamed according to their ascending chromosomal coordinates. Molecular weights and theoretical isoelectric points (pI) were predicted using the ExPASy ProtParam tool (https://www.expasy.org/). WoLF PSORT was used for subcellular localization prediction (https://wolfpsort.hgc.jp/).

### 2.2 Phylogenetic analysis

In this study, all protein sequences of the *CP12* gene family were aligned using the MEGA software (version 11.0) (Tamura et al., 2021), with alignment parameters using the default values. Based on the alignment results, a maximum likelihood (ML) method was used to construct a phylogenetic tree with 1000 bootstraps (Tayyab et al., 2025). The online tool iTOL (https://itol.embl.de/) was then used to beautify the phylogenetic tree.

### 2.3 Integrated visualization of domain, motif, and phylogenetic analyses

The conserved CP12 domain was further verified using the Conserved Domain Database (CDD) via the NCBI CD-Search tool (Marchler-Bauer et al., 2017). Conserved motifs were identified with the Meme Suite (v5.5.8) (Bailey et al., 2015). Together with the phylogenetic tree generated in MEGA, these results were integrated and visualized using TBtools.

### 2.4 Synteny analysis

To understand the evolutionary pattern of the CP12 gene family, this study performed collinearity analysis between *G. hirsutum* and three other cotton species, along with analysis within the four species. The protein sequences, coding sequences, and genome annotation files for all four species were renamed. Collinearity analysis was conducted using the One Step MCScanX function in TBtools-II software (Wang et al., 2012). Visualization included the Dual Synteny Plot for comparisons between two species, the Multiple Synteny Plot for multi-species comparisons, and the Advanced Circos function for within-species collinearity. The Ka/Ks ratio is often used to identify the type of selective pressure a gene is subject to during evolution. Ka/Ks > 1 indicates positive selection, Ka/Ks = 1 indicates neutral evolution, and Ka/Ks < 1 indicates purifying selection (Zhang, 2022). In this study, we used the Nei-Gojobori method (Jukes-Cantor correction method) built into the “Simple Ka/Ks Calculator” in the TBtools-II tool to calculate Ka and Ks values.

### 2.5 Analysis of Cis-acting regulatory elements

Members of the CP12 protein gene family were identified and extracted from the genomes of the study species to identify key targets for subsequent analysis. Using the PlantCARE (http://bioinformatics.psb.ugent.be/webtools/plantcare/html/) online database, predictive analysis of cis-acting elements within the 2000 base pair upstream regions of these family member genes was conducted to identify potential regulatory elements. A systematic statistical analysis of the type, number, and distribution of cis-acting elements within each CP12 family member was performed to establish a comprehensive element information dataset. The statistical results were visualized using RStudio and TBtools, visualizing the overall distribution of different cis-acting elements within the CP12 family members and the differences in element composition between different members.

### 2.6 Transcriptome sequencing and data analysis

To fully reflect real-world field data, this experiment used the *G. hirsutum* variety ‘Jinmian 202’, planted in a standard experimental field in the spring of 2024. Regular irrigation was used to minimize the impact of other abiotic stresses. Experimental samples were collected on July 30, 2024, when the maximum temperature reached 38 °C or above for three consecutive days. Tissues collected included flowers, leaves, and buds, and were divided into a high-temperature treatment group (GW) and a normal-temperature control group (CK). The high-temperature group refers to samples collected at 17:00 on the same day when the temperature was 38 °C, while the normal-temperature group refers to samples collected at 9:00 on the same day when the temperature was 28 °C. The cumulative duration of natural heat stress was greater than 6 hours. Flower, bud, and leaf tissues were collected under both conditions, resulting in six treatment combinations. Three independent biological replicates were performed for each treatment combination, resulting in a total of 18 samples. All samples were immediately snap-frozen in liquid nitrogen to maintain RNA integrity. Total RNA was extracted from all 18 samples and submitted to the company for transcriptome sequencing. The sequencing results were quality controlled using FASTQC and trimmed using Trimmomatic (Bolger et al., 2014). The trimmed fragments were then aligned to the reference genome using Hisat2 (Kim et al., 2019). The aligned reads were assigned to genes using featureCounts (Liao et al., 2014) to generate a raw count matrix. Differential expression analysis between GW and CK groups for each tissue was performed using the DESeq2 package (Love et al., 2014) in R.Genes with an adjusted p-value (padj) < 0.05 and absolute log2 fold change |log2FC| > 1 were identified as differentially expressed. For visualization of expression patterns, the FPKM indicator was used to quantify gene expression levels, and the pheatmap package in R was used to generate a heat map of gene expression. All transcriptome sequencing data are available from the corresponding author.

### 2.7 Quantitative real-time PCR analysis

To validate the transcriptome sequencing results, quantitative real-time PCR (qRT-PCR) was performed on selected *CP12* genes. Each sample comprises three technical replicates. Total RNA was extracted from all 18 samples using the RNAprep Pure Plant Kit (Tiangen, China) according to the manufacturer’s instructions. RNA quality and concentration were verified using a NanoDrop spectrophotometer. First-strand cDNA was synthesized from 1 μg of total RNA using the PrimeScript RT reagent Kit with gDNA Eraser (TaKaRa, Japan) to eliminate genomic DNA contamination.

Gene-specific primers for target *CP*12 genes were designed using Primer Premier 5.0 software, with the cotton UBQ7 gene serving as the internal reference control (Tu et al., 2007). qRT-PCR reactions were carried out using TB Green Premix Ex Taq II (TaKaRa, Japan) on a QuantStudio 5 Real-Time PCR System (Applied Biosystems, USA). Each 10 μL reaction mixture contained 5 μL of TB Green Premix Ex Taq II, 0.3 μL of each primer (10 μM), and 4.4 μL of cDNA template (diluted 100-fold). The thermal cycling protocol included initial denaturation at 95 °C for 120 s, followed by 40 cycles of 95 °C for 15 s and 60 °C for 30 s. A melting curve analysis was performed at the end of each amplification to verify reaction specificity. The presence of a single, sharp peak for all primer pairs confirmed the amplification of specific targets and the absence of primer-dimers or other non-specific products. Relative expression levels were calculated using the 2^(-ΔΔCt) method (Livak and Schmittgen, 2001).

### 2.8 Analysis of physiological and biochemical indices

To assess the effects of heat stress on cotton physiology, we measured a series of key physiological and biochemical indicators. First, we used the LI-6400/XT Portable Photosynthesis System to measure net photosynthetic rate (Pn), stomatal conductance (Cond), transpiration rate (Tr), and intercellular carbon dioxide concentration (Ci) from 9:00 to 17:00 (https://www.licor.com/support/LI-6400/home.html). Antioxidant enzyme activity and oxidative damage markers were measured using commercially available kits provided by Beijing solarbio science & Technology co, Ltd., including the catalase (CAT) activity assay kit (BC0205), the peroxidase (POD) activity assay kit (BC0095), and the malondialdehyde (MDA) content assay kit (BC0025) (Afiyanti and Chen, 2014; Wahid and Khaliq, 2015; Hao et al., 2023). Tissue samples were collected from flowers, leaves, and buds, and procedures were performed according to the kit instructions. Three technical replicates were performed for each sample.

## 3 Result

### 3.1 Genome-wide identification and basic characterization of the *CP12* gene family in *Gossypium* species

After blastp comparison and HMMER search, a total of 30 *CP12* genes were screened out in the four cotton species, of which 11, 10, 5, and 4 *CP12* genes were screened out in *G. hirsutum, G. barbadense, G. arboreu*m, and *G. raimondii*, respectively. The number of *CP12* genes contained in the two tetraploid cottons is about twice that of their diploid ancestors.

**Figure 1:**
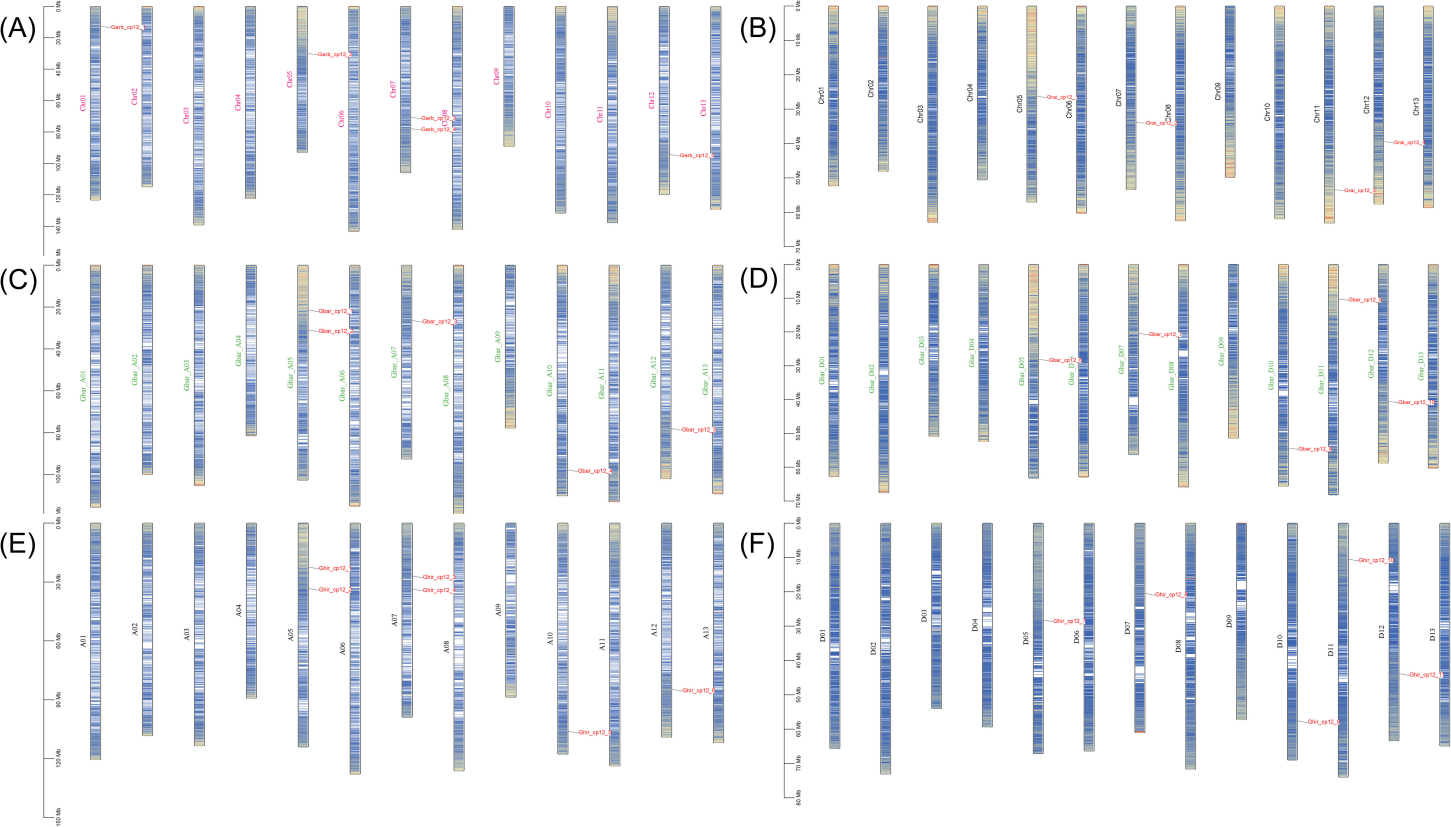
Chromosomal distribution of *CP12* genes in four *Gossypium* species. Chromosomal locations of CP12 genes are shown according to their reference genome assemblies. **(A)** *G. arboreum*, **(B)** *G. raimondii*, **(C)** *G. barbadense* at subgenome, **(D)** *G. barbadense* Dt subgenome, **(E)** *G. hirsutum* At subgenome, and **(F)** *G. hirsutum* Dt subgenome.

Prediction results indicated that all CP12 proteins are located in the chloroplast, confirming their role in photosynthesis. Further analysis showed considerable diversity in protein traits. Protein lengths ranged from 87 to 187 amino acids, with molecular weights between 10.05 and 20.90 kDa. Most proteins had acidic isoelectric points (pI 4.41-5.78). Based on instability index values (40), most were classified as unstable, which is typical for regulatory proteins that are rapidly turned over. Aliphatic indices (54.56-80.56) suggested moderate stability at higher temperatures, and negative GRAVY values (-0.994 to -0.41) indicated that all proteins are hydrophilic and likely soluble in cell environments (**Table 1**). All genes were renamed based on their chromosomal locations (e.g., Ghir_CP12_1 to Ghir_CP12_11) for easier reference in later studies (**Figure 1**).

**Table 1:** Physicochemical properties and subcellular localization of CP12 proteins in four *Gossypium species* and *Arabidopsis thaliana*.

Gene_name	Gene_ID	accession numbers(NCBI)	Number ofAminoAcid	MolecularWeight	TheoreticalpI	InstabilityIndex	AliphaticIndex	GrandAverageofHydropathicity	Predictedlocation
AT1G76560.1	AT_cp12_1	NP_565134.1	134	15448.53	4.93	64.51	69.85	-0.688	chlo
AT2G47400.1	AT_cp12_2	NP_566100.2	124	13487.13	4.86	37.21	70	-0.5	chlo
AT3G62410.1	AT_cp12_3	NP_191800.1	131	14166.75	4.82	39.8	67.1	-0.592	chlo
Garb_01G007500.1	Garb_cp12_1	XP_012447237.1	87	10047.47	5.08	45.24	62.76	-0.897	chlo
Garb_05G027640.1	Garb_cp12_2	XP_012447237.1	136	15747.82	5.28	48.02	54.56	-0.902	chlo
Garb_07G012430.1	Garb_cp12_3	MBA0749801.1	87	10128.41	4.86	49.89	62.76	-0.994	chlo
Garb_07G014360.1	Garb_cp12_4	KAK5818774.1	126	14091.65	4.64	54.27	71.35	-0.752	chlo
Garb_12G016480.1	Garb_cp12_5	XP_016729496.1	122	13413.07	4.82	35.98	67.95	-0.707	chlo
Gbar_A05G022230.1	Gbar_cp12_1	XP_012447237.1	94	10897.2	4.41	55.05	64.26	-0.901	chlo
Gbar_A05G027890.1	Gbar_cp12_2	XP_012447237.1	136	15695.74	5.26	43.8	54.56	-0.892	chlo
Gbar_A07G013580.1	Gbar_cp12_3	KAK5818774.1	126	14049.61	4.64	52.92	70.56	-0.75	chlo
Gbar_A10G020140.1	Gbar_cp12_4	XP_016677020.1	126	14172.1	5.78	55.52	63.49	-0.733	chlo
Gbar_A12G013550.1	Gbar_cp12_5	XP_016729496.1	122	13387.03	4.82	32.99	68.77	-0.679	chlo
Gbar_D05G028770.1	Gbar_cp12_6	XP_012447237.1	136	15629.62	5.27	43.26	54.56	-0.949	chlo
Gbar_D07G014030.1	Gbar_cp12_7	MBA0790727.1	126	14006.56	4.73	51.62	64.37	-0.774	chlo
Gbar_D10G020350.1	Gbar_cp12_8	MBA0749801.1	135	15302.32	5.35	59.27	65.7	-0.73	chlo
Gbar_D11G011980.1	Gbar_cp12_9	XP_016709017.2	128	14053.66	5.5	52.34	62.5	-0.82	chlo
Gbar_D12G013560.1	Gbar_cp12_10	XP_016729496.1	161	17812.39	5.74	37.72	80.56	-0.41	chlo
Ghir_A05_G02374.t1	Ghir_cp12_1	XP_012447237.1	94	10979.35	4.53	49.23	65.32	-0.924	chlo
Ghir_A05_G02987.t1	Ghir_cp12_2	XP_012447237.1	136	15695.74	5.26	43.8	54.56	-0.892	chlo
Ghir_A07_G01533.t1	Ghir_cp12_3	KAK5818774.1	186	20798.52	5.45	51.29	76.13	-0.579	chlo
Ghir_A07_G01721.t1	Ghir_cp12_4	TYI28419.1	94	10907.2	4.64	45.21	61.17	-0.965	chlo
Ghir_A10_G02220.t1	Ghir_cp12_5	XP_016677020.1	126	14172.1	5.78	55.52	63.49	-0.733	chlo
Ghir_A12_G01525.t1	Ghir_cp12_6	XP_016729496.1	122	13413.07	4.82	35.98	67.95	-0.707	chlo
Ghir_D05_G02994.t1	Ghir_cp12_7	XP_012447237.1	136	15629.62	5.27	43.26	54.56	-0.949	chlo
Ghir_D07_G01532.t1	Ghir_cp12_8	MBA0790727.1	187	20897.56	5.64	50.44	71.02	-0.612	chlo
Ghir_D10_G02232.t1	Ghir_cp12_9	XP_016677020.1	135	15360.36	5.22	58.36	64.96	-0.769	chlo
Ghir_D11_G01236.t1	Ghir_cp12_10	XP_016709017.2	128	14053.66	5.5	52.34	62.5	-0.82	chlo
Ghir_D12_G01613.t1	Ghir_cp12_11	XP_016729496.1	122	13415.04	4.82	35.52	65.57	-0.752	chlo
Grai_05G030300.1	Grai_cp12_1	XP_012447237.1	136	15629.62	5.27	43.26	54.56	-0.949	chlo
Grai_07G014410.1	Grai_cp12_2	MBA0790727.1	126	14061.58	4.73	51.99	64.37	-0.796	chlo
Grai_11G027530.1	Grai_cp12_3	XP_017630085.1	128	14053.66	5.5	52.34	62.5	-0.82	chlo
Grai_12G016240.1	Grai_cp12_4	XP_016729496.1	122	13415.04	4.82	35.52	65.57	-0.752	chlo

### 3.2 Phylogenetic analysis and evolutionary relationships of CP12 proteins

**Figure 2:**
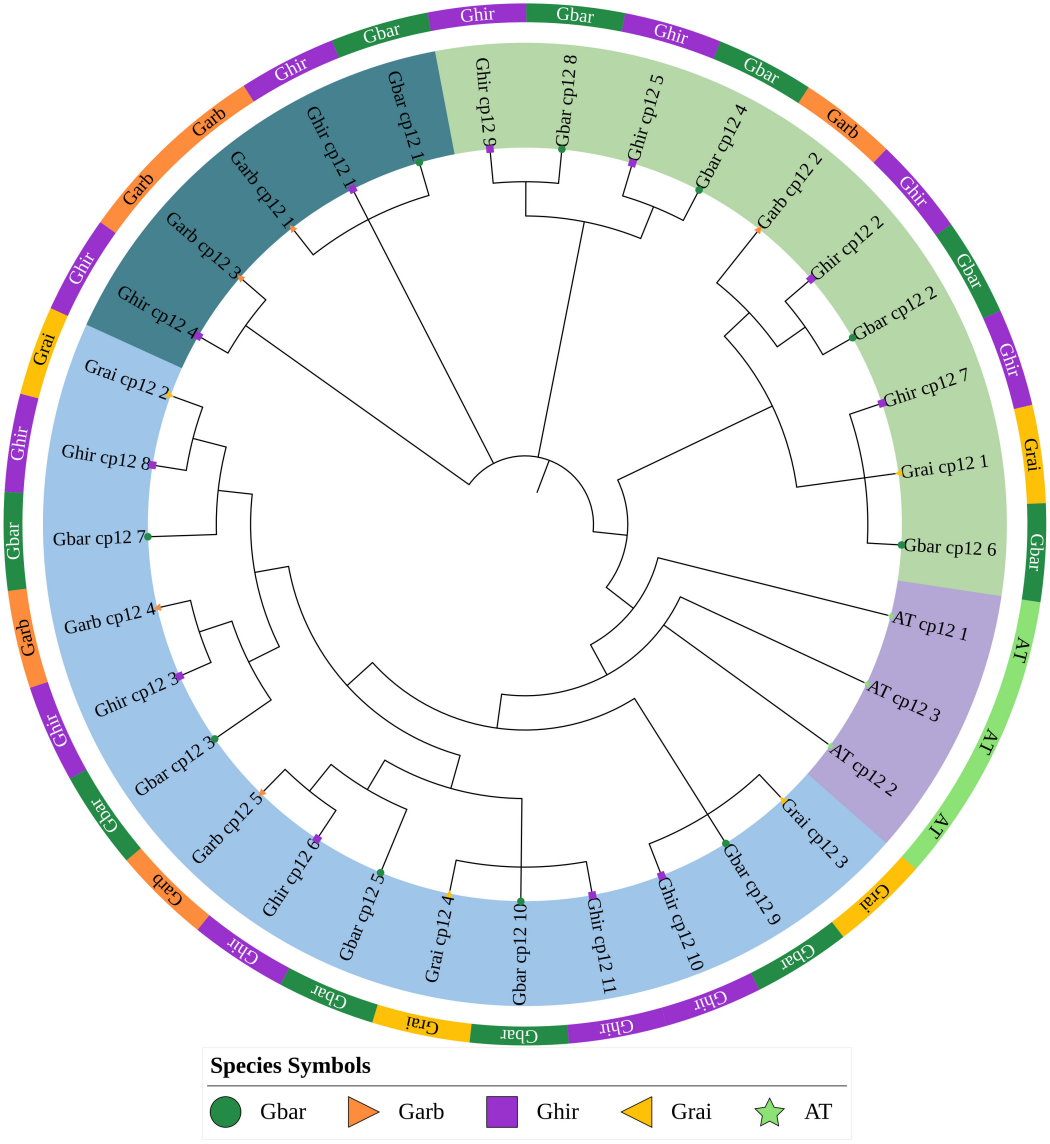
Phylogenetic analysis of CP12 proteins from *Gossypium* species and Arabidopsis thaliana. The maximum-likelihood tree was constructed using MEGA 11 with 1,000 bootstrap replicates. The three A. thaliana CP12 proteins serve as the outgroup. Cotton CP12 proteins are grouped into three major clades (I, II, and III), highlighted by colored backgrounds.

After obtaining the results of multiple sequence alignment, phylogenetic tree analysis was performed (**Figure 2**). The results of phylogenetic analysis showed that the 30 *CP12* genes of the four cotton species can be divided into three subfamilies, of which Clade I contains 15 genes, Clade II contains 10 genes, and Clade III contains five genes. The three *AtCP12* genes form an outgroup alone, which indicates that the *CP12* genes of cotton and *Arabidopsis thaliana* may have undergone significant differentiation.

### 3.3 Conserved motif and gene structure analysis

**Figure 3:**
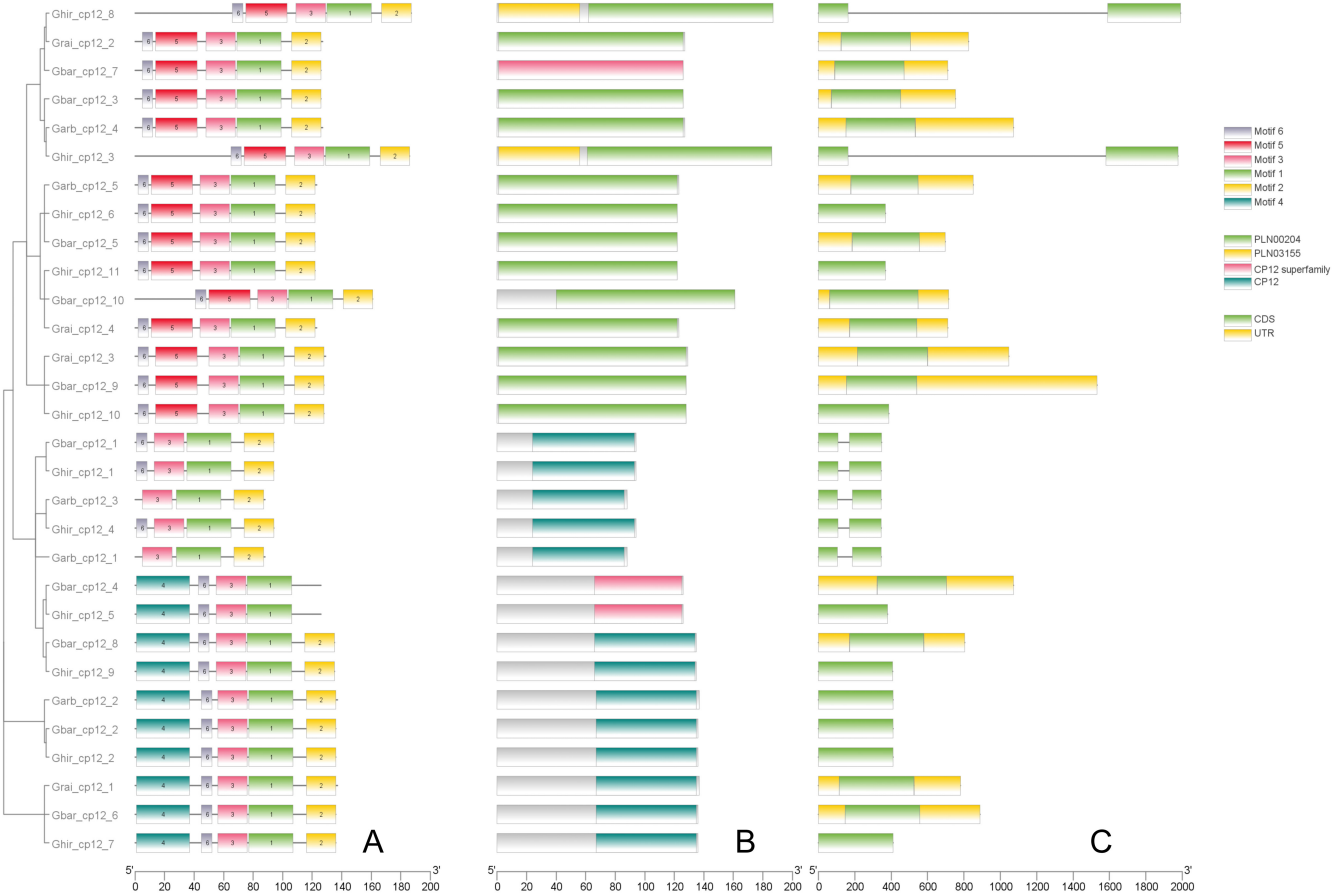
Structural and functional domains of CP12 genes in *Gossypium* species. **(A)** Distribution of conserved protein motifs identified by MEME Suite (Motifs 1–6). Each motif is represented by a colored box. **(B)** Conserved CP12 domains as predicted by NCBI CDD. **(C)** Exon–intron structures of *CP12* genes.

After a comprehensive analysis of conserved motifs and gene structure (**Figure 3**), phylogenetic classification was further supported (**Figure 2**). Meme analysis showed that all Clade I members contained conserved motifs 1, 2, 3, 5, and 6. All Clade II members contained conserved motifs 1, 3, 4, and 6, while all Clade III members contained conserved motifs 1, 2, and 3. In addition, all Clade I members contained at least one CDS region, all Clade II members contained only one CDS region, and all Clade III members contained two CDS regions and lacked UTR regions.

### 3.4 Synteny analysis of *CP12* genes in *Gossypium* species

**Figure 4:**
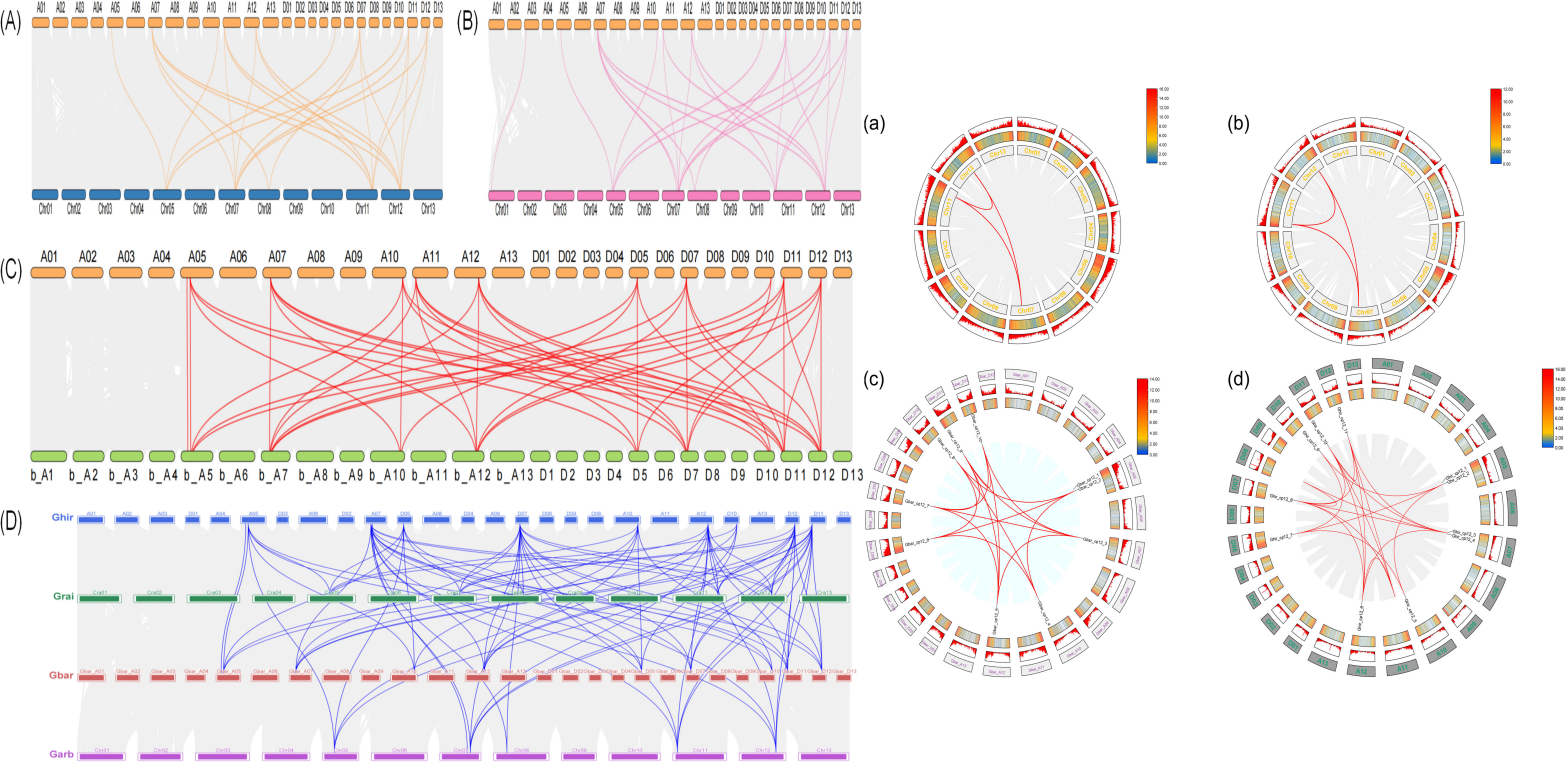
Synteny analysis of *CP12* genes in *Gossypium* species. Left panel: Inter-genomic synteny analysis between *G. hirsutum* and related species. **(A)** *G. hirsutum* vs. *G. raimondii*, **(B)** *G. hirsutum* vs. *G. arboreum*, **(C)** *G. hirsutum* vs. *G. barbadense*, and **(D)** summary of syntenic relationships among the four species. Gray lines indicate collinearity among all genes, and colored lines highlight syntenic CP12 pairs. Right panel: Intra-genomic synteny analysis. Circos plots illustrate collinear relationships within **(a)** G. raimondii, **(b)** G. arboreum, **(c)** G. barbadense, and **(d)** *G. hirsutum*. Red lines indicate homologous CP12 gene pairs. Tetraploid cottons possess more syntenic pairs *(G. barbadense*: 16; *G. hirsutum*: 21) than diploid ancestors (3 pairs each).

Results from interspecific synteny analysis (**Figure 4**, left, A-D) showed that the *CP12* gene family contains 24 orthologous pairs between *G. hirsutum* and *G. raimondii*, with 12 pairs in the At subgenome and 12 in the Dt subgenome, displaying a relatively uniform distribution. Between *G. hirsutum* and *G. arboreum*, there are 25 orthologous pairs, including 12 in the At subgenome and 13 in the Dt subgenome. The highest number of orthologous pairs occurs between *G. hirsutum* and the tetraploid *G. barbadense* (sea island cotton), totaling 45 pairs with 24 in the At subgenome and 21 in the Dt subgenome. Additionally, *CP12* genes located on chromosome 7 (At and Dt subgenomes) of *G. hirsutum* were found to be syntenic with multiple genes in the other three cotton species, a pattern that may have contributed to the evolution of the cotton *CP12* gene family. Variation was observed in intraspecific gene duplication of *CP12* genes (**Figure 4**, right, a-d). Each of the two diploid cotton species contains only three pairs of paralogous genes. In contrast, tetraploid cotton species exhibit significantly more intraspecific and intra-subgenomic paralogous synteny, with *G. barbadense* containing 16 pairs and *G. hirsutum* 21 pairs. Consistent with the pattern seen in interspecific synteny, the most abundant *CP12* genes are also localized on chromosome 7.

### 3.5 Selection pressure analysis of *CP12* genes

**Figure 5:**
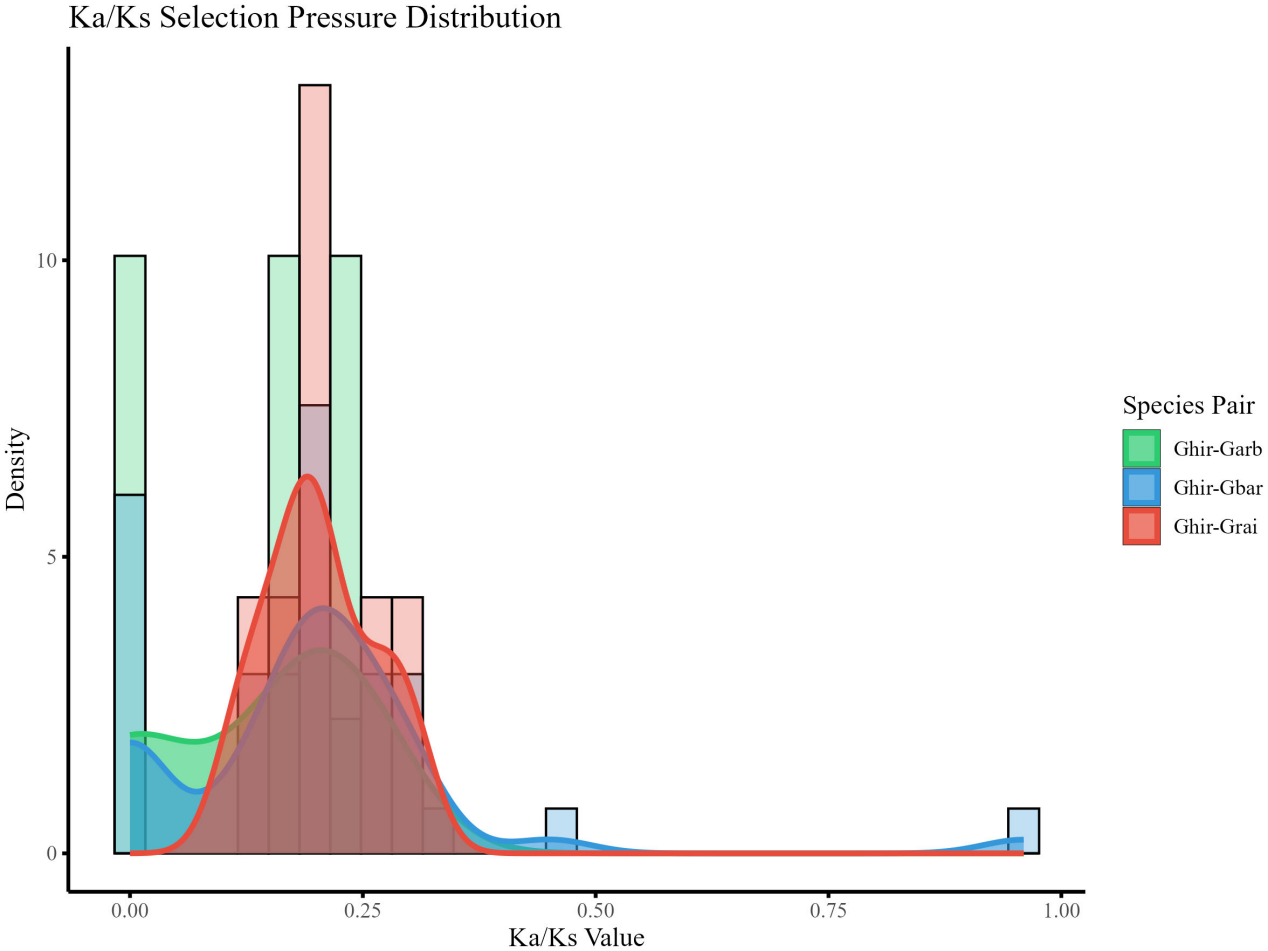
Selection pressure analysis of CP12 genes between *G. hirsutum* and related *Gossypium* species. Density distribution of the nonsynonymous/synonymous substitution rate ratio (Ka/Ks) for syntenic gene pairs between *G. hirsutum* and related species (*G. arboreum, G. barbadense, G. raimondii*). All Ka/Ks values < 1 indicate strong purifying selection during evolution.

To assess the evolutionary constraints on the *CP12* gene family, a selection pressure analysis was conducted (**Figure 5**). All Ka/Ks ratios were found to be below 1.0, indicating that the CP12 gene family has undergone strong purifying selection in the *genus Gossypium*. Specifically, the maximum Ka/Ks ratio was 0.45, while the minimum was 0. Further comparison of the protein sequences of genes with a Ka/Ks ratio of 0 revealed that they share identical amino acid sequences. From these observations, it can be inferred that the *CP12* gene family in *Gossypium* is a gene family with highly conserved functions and subject to strong purifying selection, playing an irreplaceable role in the biological processes of cotton species.

### 3.6 Analysis of Cis-acting regulatory elements

**Figure 6:**
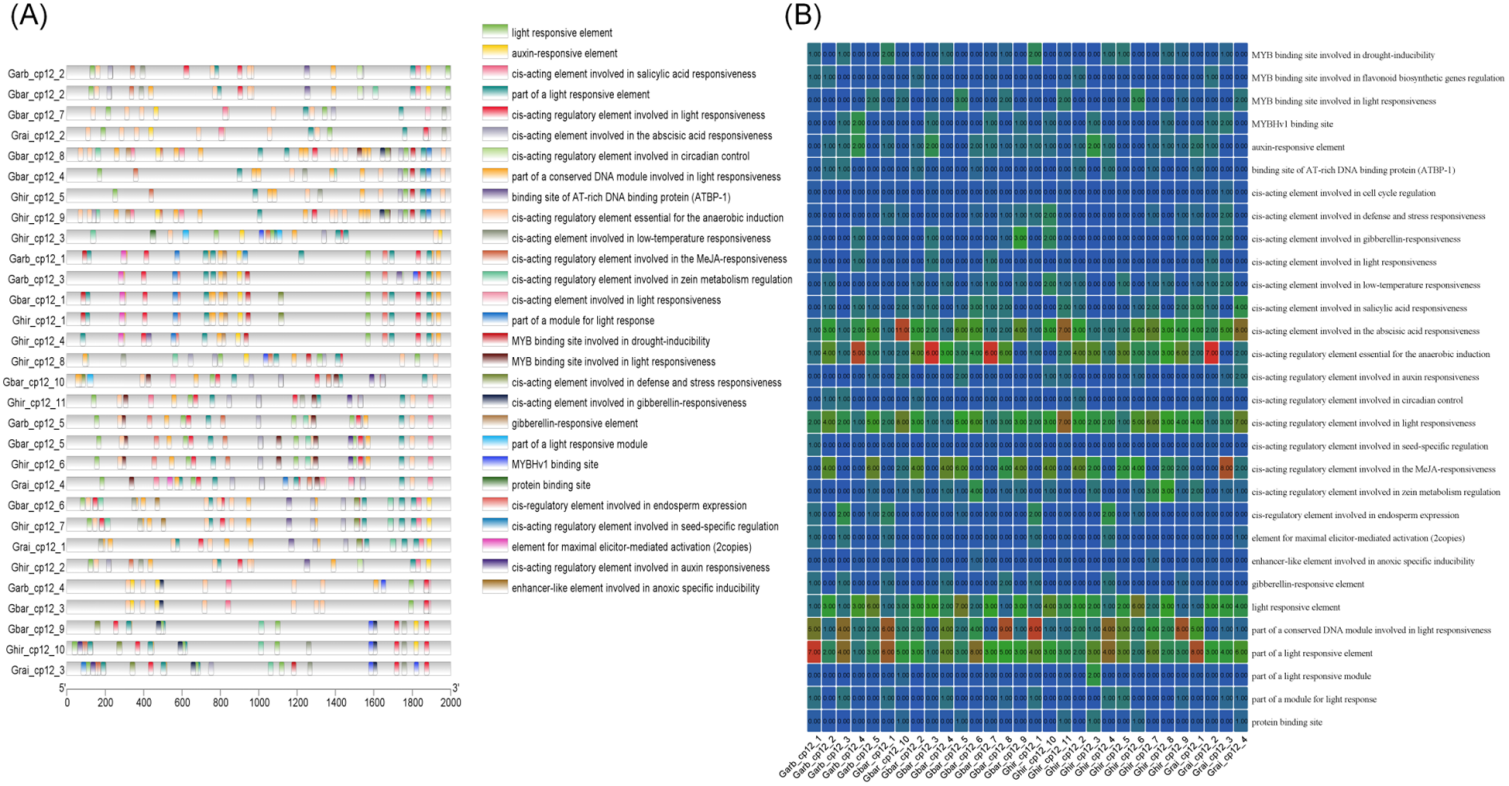
Analysis of cis-regulatory elements in the promoters of *G.hirsutum* CP12 genes. **(A)** Distribution and composition of cis-acting elements within the 2000 bp upstream promoter regions. **(B)** Heatmap showing the abundance of stress-responsive elements.

Analysis of cis-acting elements in the promoter regions of the *CP12* gene family revealed a total of 30 predicted cis-acting elements across all family members (**Figure 6**). Of these 30 elements, light-responsive elements were the most numerous, with eight, followed by plant hormone and transcription factor binding site elements, with six, and then abiotic stress-related elements, with five. The distribution of these elements across species exhibited specific characteristics (**Supplementary Figure 1**): 20 cis-acting elements were shared by the four cotton species; one unique element, a cis-acting regulatory element involved in seed-specific regulation, was found in *G.arboreum*; one unique element, a cis-acting regulatory element involved in cell cycle regulation, was found in *G.raimondii;* no unique cis-acting elements were detected in *G.hirsutum* and *G.barbaadense*.

### 3.7 Expression profiling of G. hirsutum *CP12* genes under heat stress

**Figure 7:**
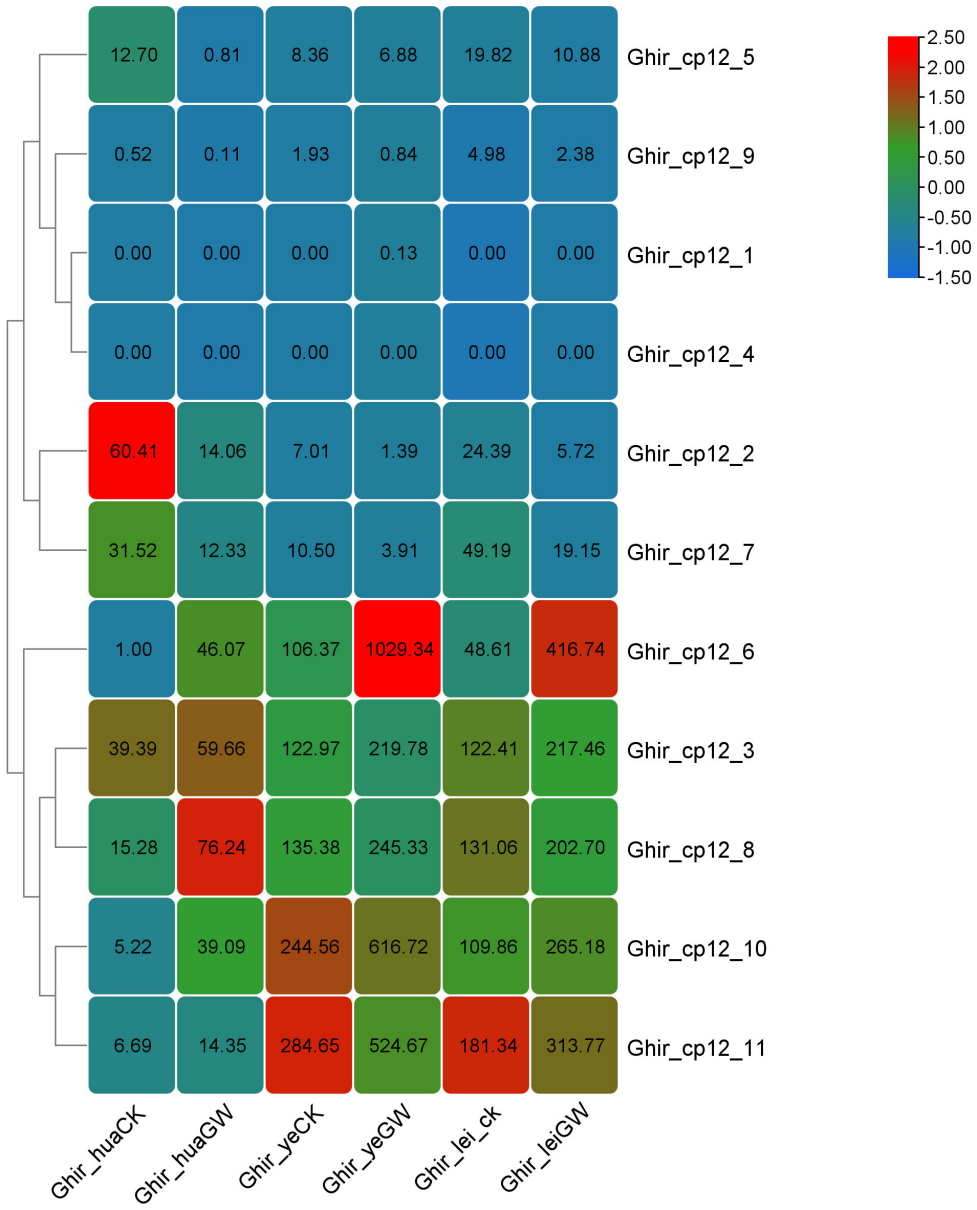
Expression patterns of CP12 genes in G. hirsutum under normal and heat stress conditions. Heatmap of CP12 gene expression in flowers, leaves, and buds under control (28 °C) and heat stress (38 °C) conditions. Blue indicates low expression, and red indicates high expression. Expression values are based on RNA-seq (FPKM) data.

Analysis of the expression patterns of different *CP12* subfamily genes in various tissues of *G. hirsutum*, combined with FPKM expression levels, revealed a noteworthy pattern (**Supplementary Table 3**). We defined significantly differentially expressed genes using a threshold of |log2FC| > 1 and padj < 0.05 (**Figure 7**). Under heat stress, genes from Clade I showed an upregulated expression trend in flowers, leaves, and buds. The Ghir_CP12_10 gene exhibited a particularly significant increase, with expression levels approximately 10-fold higher in flowers, leaves, and buds after heat treatment. The expression levels of the other four Ghir genes belonging to Clade I also increased significantly.

Genes from Clade II exhibited the opposite trend, a significant down-regulation under heat stress. For example, Ghir_CP12_2 showed an approximately 5-fold decrease in expression in flowers, leaves, and buds after heat treatment. Clade III genes, on the other hand, exhibited relatively stable expression, with slight fluctuations in expression levels under heat stress.

### 3.8 Validation of RNA-seq expression patterns by qRT-PCR

**Figure 8:**
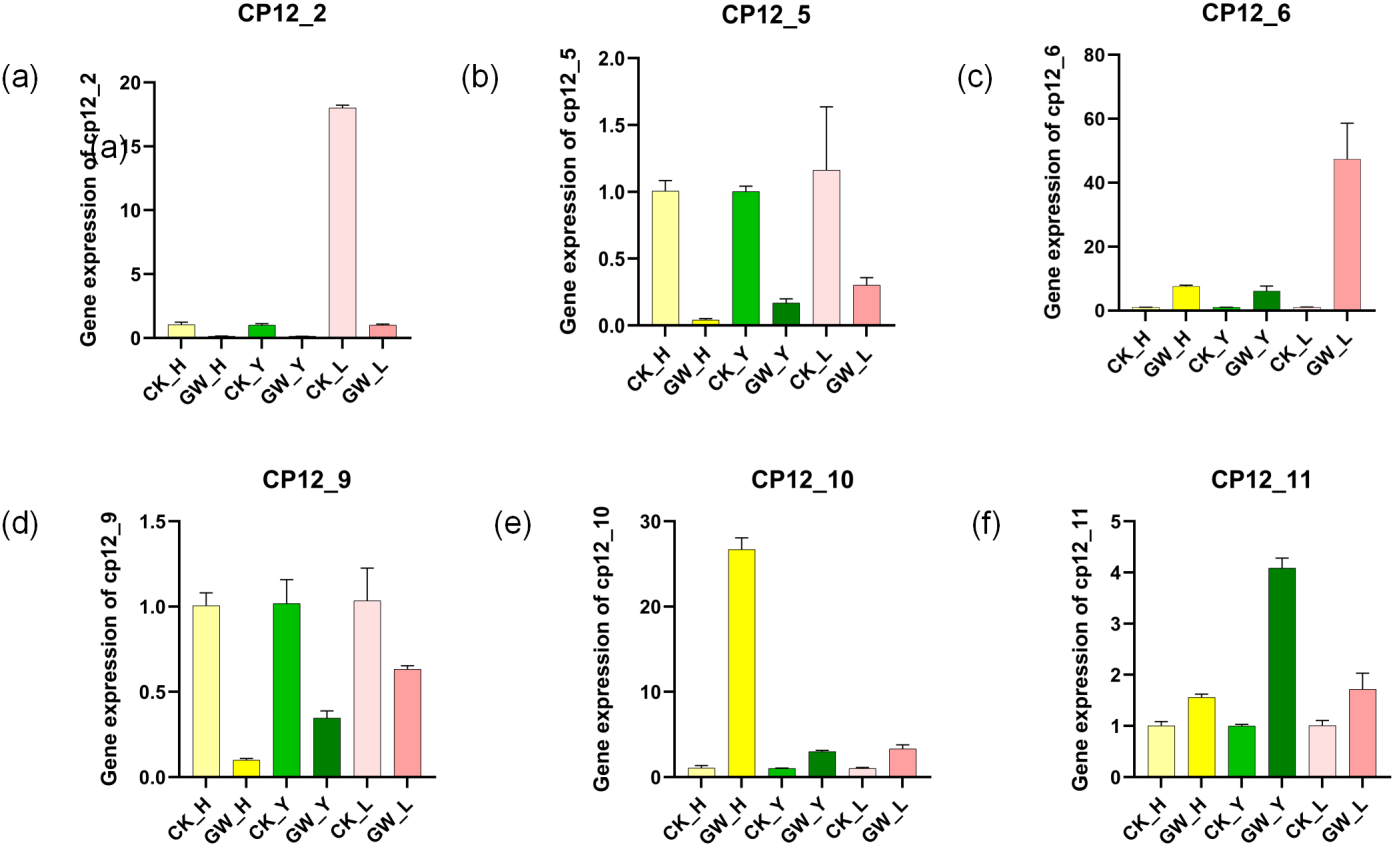
Validation of*G hirsutum CP12* gene expression patterns under heat stress by qRT-PCR. Relative expression levels of six selected CP12 genes representing different clades in flowers, leaves, and buds under control (28 °C) and heat stress (38 °C) conditions. Each value represents the mean ± SE of three biological replicates. **(a)** Ghir_CP12_2 **(b)** Ghir_CP12_5 **(c)** Ghir_CP12_6 **(d)** Ghir_CP12_9 **(e)** GhirCP_12_10 **(f)** Ghir_CP12_11 The relatively low expression levels of some genes in specific tissues resulted in small variation among replicates, making error bars less apparent. For instance, Ghir_CP12_2 exhibited very weak expression in flowers and leaves under heat stress conditions.

To validate the transcriptome results, we randomly selected six *G. hirsutum CP12* genes for qPCR validation in three tissues: flowers, leaves, and buds (**Supplementary Table 2**). These analyses were performed under both normal temperature (28 °C) and heat stress (38 °C) conditions, ensuring consistency with the transcriptome analysis (**Figure 8**).

The results showed that the qPCR results were highly consistent with the expression trends in the transcriptome data. Under heat stress conditions, the expression of Ghir_CP12_10 increased significantly in flowers, and the expression of Ghir_CP12_6 in buds was also significantly higher than in the control group. Ghir_CP12_11 also showed a significant increase in expression. Conversely, the expression of Ghir_CP12_5 and Ghir_CP12_9 was significantly downregulated in flowers, leaves, and buds.

### 3.9 Photosynthetic characteristics and antioxidant physiological responses of cotton

**Figure 9:**
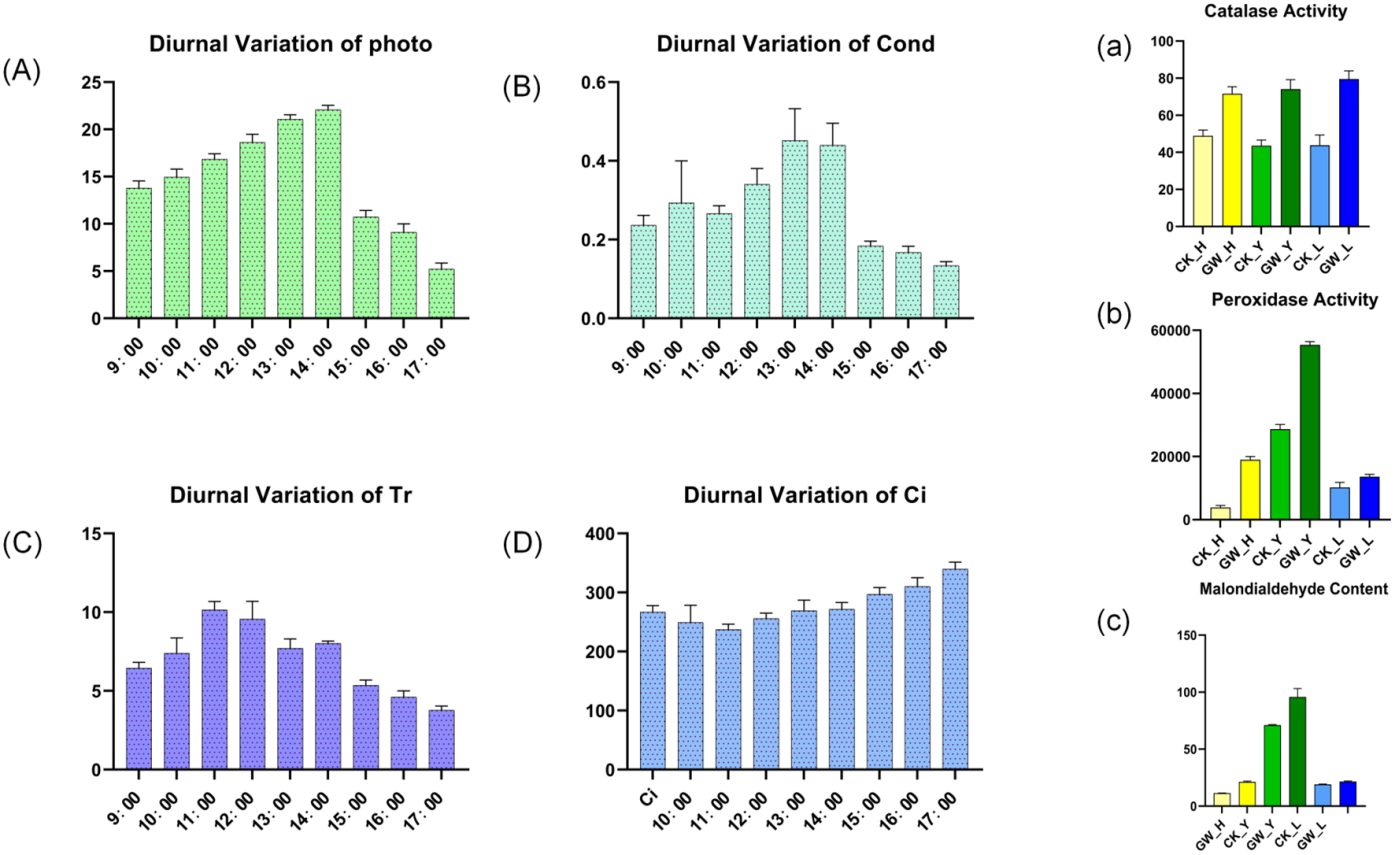
Photosynthetic and physiological responses of cotton under heat stress. Left panel: Diurnal variation of photosynthetic parameters. **(A)** net photosynthetic rate (Pn), **(B)** stomatal conductance (Gs), **(C)** transpiration rate (Tr), and **(D)** intercellular CO_2_ concentration **(Ci)** under control and heat stress conditions. Right panel: Physiological and biochemical indicators **(a)** catalase (CAT) activity, **(b)** peroxidase (POD) activity, and **(c)** malondialdehyde (MDA) content in leaves, flowers, and buds.

Measurements of photosynthetic parameters revealed a single-peaked curve for Pn, rising continuously from 9:00 AM to 2:00 PM, reaching a maximum of 22.4 μmol·m^-^²·s^-^¹ at 2:00 PM. A significant decrease in photosynthetic rate occurred between 2:00 PM and 3:00 PM, before reaching a minimum of 7.4 at 5:00 PM. Gs and Pn showed highly similar trends, as did Tr and Gs, but Ci showed some differences, exhibiting a brief decline followed by a steady increase (**Figure 9**, left, A-D). Furthermore, under high temperatures, CAT activity increased in all plant species, with a particularly pronounced increase in leaves. POD activity responded strongly to high temperatures, showing a highly significant change in leaves. MDA content generally increased after high-temperature stress (**Figure 9**, right, a-c).

## 4 Discussion

Our study addresses the prior knowledge gap regarding CP12 in allotetraploid cotton by revealing significant expansion and functional divergence of the *CP12* gene family. Overall, our research findings are as follows.

Whole-genome analysis of the *CP12* gene family across four *Gossypium* species revealed significant expansion and diversification, forming three well-supported phylogenetic clades. This pattern is comparable to the three CP12 clades identified in *Arabidopsis thaliana* (Groben et al., 2010). Unlike the high functional redundancy in AtCP12 isoforms, their cotton counterparts exhibited clear divergence under heat stress, with Clade I being upregulated, Clade II being down-regulated, and Clade III remaining stable. Such expression divergence suggests subfunctionalization following gene duplication, potentially reflecting evolutionary adaptation to the elevated temperatures typical of cotton-growing regions.

In floral tissues, all five members of Clade I were significantly upregulated in response to heat stress, indicating a conserved regulatory role in thermal protection. This finding is consistent with functional studies in other species, which demonstrate that CP12 protects photosynthetic enzymes from oxidative and thermal damage by stabilizing GAPDH and PRK. For instance, in *Chlamydomonas reinhardtii*, CP12 prevents the thermal aggregation of GAPDH, maintaining approximately 80% enzyme activity at 43 °C, whereas the absence of CP12 results in complete loss of activity (López-Calcagno et al., 2014). In cyanobacteria, salt and osmotic stress induce the up-regulation of CP12-C, while dark aerobic conditions drive a fourfold increase in CP12-N-CBS expression (Stanley et al., 2013). Moreover, overexpression of CP12 in the tropical legume *Stylosanthes guianensis* enhances biomass accumulation by 30% under low temperatures (Gérard et al., 2022a). Collectively, these findings suggest that cotton Clade I genes have retained an ancestral protective role for photosynthetic enzymes and redox balance, with possible specialization for heat adaptation.

In contrast, Clade II genes were generally down-regulated under heat stress, implying an alternative role in carbon partitioning and energy conservation during adverse conditions. Previous research in tobacco demonstrated that suppressing CP12 expression disrupts carbon allocation. This suppression triggered a shift in carbon flow, enhancing its diversion toward cell wall components and malate while reducing starch and soluble carbohydrate synthesis. Expression of APS reductase and sulfate transporters also increased, indicating metabolic reprogramming in response to stress (Howard et al., 2011). Similarly, in cotton, the repression of Clade II genes may represent an adaptive mechanism that redirects carbon flux from growth to stress mitigation and cellular protection.

In contrast, Clade III genes maintain relatively stable expression levels under heat stress, suggesting that they may only have a basic maintenance function in the three parts of the flower, leaf, and bud. This view is supported by their streamlined gene structure, which is characterized by the lack of untranslated regions (UTRs) and the presence of only core conserved motifs. This is consistent with observations in *Arabidopsis thaliana*, where Clade III genes are expressed only in non-photosynthetic tissues such as roots, rather than in photosynthetic tissues (Sun et al., 2009).

Physiological data from this study further support the molecular findings. Heat stress resulted in a decline in photosynthetic rate and a concomitant increase in antioxidant enzyme activity. The up-regulation of Clade I *CP12* genes correlated with enhanced peroxidase activity and elevated malondialdehyde (MDA) levels, consistent with their involvement in mitigating oxidative stress. In tobacco, the inhibition of CP12 expression resulted in a significant increase in polyphenol oxidase (PPO) transcription. CP12-deficient cyanobacteria accumulated 1.7 times higher levels of ROS compared with the wild type (Howard et al., 2011). Similarly, CP12 knockout mutants exhibited upregulation of ROS-scavenging proteins and six heat shock proteins (HSPs), suggesting that an enhanced antioxidant defense system compensates for oxidative stress caused by CP12 deficiency (Gérard et al., 2022a).

While the correlations between gene expression and physiological parameters are compelling, this study remains primarily descriptive in nature. Future functional validation is required to establish causality, particularly through gene manipulation approaches such as virus-induced gene silencing (VIGS) or CRISPR-Cas9 knockout of *CP12* genes. Although Clade III genes were described as housekeeping in this study, their potential roles under stress remain unexplored and warrant further investigation. We hypothesize that knockdown of heat-induced Clade I genes would increase plant sensitivity to heat stress, leading to stronger photoinhibition, greater oxidative damage, and potential yield penalties. Additionally, since heat stress treatments were conducted under natural field conditions, environmental variability may have influenced the results. Therefore, future studies should combine controlled-environment experiments with metabolomic and proteomic analyses to clarify the mechanistic basis of CP12-mediated stress responses.

## 5 Conclusion

In this study, we identified and characterized the *CP12* gene family in four Gossypium species, revealing its expansion into three clades mainly driven by genome duplications. Expression analyses revealed clade-specific heat stress responses, with Clade I genes being strongly upregulated and most Clade II genes repressed, suggesting functional divergence. Physiological data supported these transcriptional trends. Our findings highlight Clade I *of CP12* genes as key regulators of thermotolerance, providing potential targets for improving heat resilience in cotton.

## Data Availability

The data presented in the study are deposited in the NCBI Sequence Read Archive (SRA) under BioProject accession number PRJNA1330244. The data will be publicly available at the following link after the release date: https://www.ncbi.nlm.nih.gov/sra/PRJNA1330244.

## Supplementary material

Supplementary Data Sheet 1 Protein sequences of the CP12 gene family.

Supplementary Table 1 Protein sequences of the CP12 gene family.

Supplementary Table 2 Primer sequences used for qRT-PCR analysis of CP12. The table includes gene identifiers, primer forward and reverse sequences, amplicon size (bp), and melting temperature (Tm).

Supplementary Table 3 Transcriptome expression profiles (FPKM values) of *G. hirsutum CP12* genes. This table provides FPKM values for each gene, mean ± SE across biological replicates, and statistical significance (padj) for comparisons between heat-stressed (GW) and control (CK) conditions.

Supplementary Figure 1 The Venn diagram illustrates the overlap of cis-acting elements identified within 2000 bp upstream of the promoter regions of CP12 genes across four Gossypium species.
