# Supplementary figures and images for: Genome-wide identification and functional characterization of the CP12 gene family in cotton reveals its critical role in heat stress response
Source: Front Plant Sci. 2025 Oct 30;16:1707567. doi: 10.3389/fpls.2025.1707567 (PMC12612862; doi:10.3389/fpls.2025.1707567)

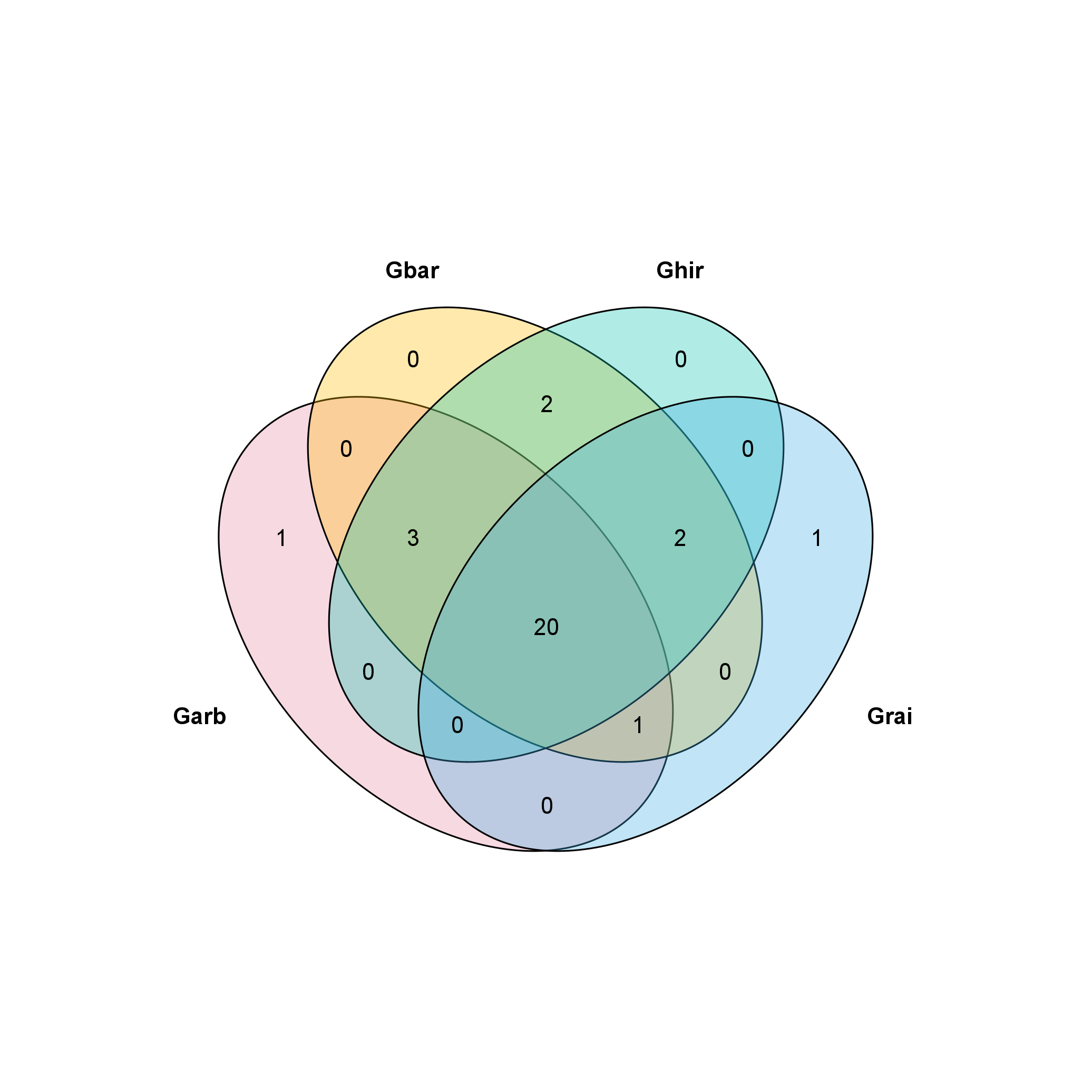

Supplement: Supplementary Data Sheet 1 — Protein sequences of the CP12 gene family. [file DataSheet1.zip › Supplementary/Supplementary Figure 1.TIFF]
